# Identifying central positional vertigo to prevent diagnostic pitfalls: A case series and review of literature

**DOI:** 10.1016/j.radcr.2026.07.009

**Published:** 2026-07-25

**Authors:** Quentin Legois, Paul Fargeas, Alexandra Weckel, Salim Ismail, Mikhael Makhoul, Pauline Nieto, Charles-Edouard Molinier, Olivier Deguine, Mathieu Marx

**Affiliations:** a ORL, Otoneurology and ORL Pediatric Department, CHU Toulouse, Toulouse, France; b UMR5549 - CerCo, CNRS, Brain and Cognition Research Center, Toulouse, France; c Institut de Formation de Masso-Kinésithérapie, PREFMS, Pôle Régional d'Enseignement et de Formation aux Métiers de la Santé, Toulouse, France; d Otology Auditory Implants and Skull Base Surgery Department, Pitié-Salpêtrière Hospital, Paris, France

**Keywords:** Central positional vertigo, Benign paroxysmal positional vertigo, Brain MRI, Repositioning maneuver, Case series

## Abstract

This case series highlights the critical role of radiological imaging in patients presenting with positional vertigo initially suggestive of benign paroxysmal positional vertigo (BPPV) but ultimately attributable to central nervous system pathology. The objective is to raise awareness among clinicians and paramedical professionals regarding the importance of early imaging when atypical clinical features are present. We report 6 cases of central positional vertigo in patients aged 18-85 years (3 men and 3 women) initially referred for physiotherapy with a presumed diagnosis of BPPV. Detailed clinical analysis revealed red flags prompting neurological evaluation and neuroimaging. Magnetic resonance imaging (MRI) played a decisive role in all cases, enabling accurate etiological diagnosis. Imaging protocols included brain MRI with T1- and T2-weighted, FLAIR, diffusion-weighted imaging with apparent diffusion coefficient mapping, susceptibility-weighted imaging when indicated, and postcontrast T1 sequences following gadolinium administration. Additional sequences such as constructive interference in steady state (CISS/FIESTA) were used when neurovascular conflict was suspected. Identified etiologies included cerebellar hemangioblastoma, vestibular migraine with radiologic findings, neurovascular compression syndrome, cerebellar atrophy, cerebellar meningioma, and midbrain ischemic stroke. MRI findings were essential for differentiating central from peripheral causes of positional vertigo and for guiding urgent management, particularly in vascular and tumoral conditions. In all cases, imaging corrected the initial misdiagnosis of peripheral vertigo and prevented inappropriate repositioning maneuvers. This case series underscores the importance of integrating clinical examination with early neuroimaging when positional vertigo presents with central warning signs. Brain MRI remains the gold standard for identifying central causes and improving patient outcomes.

## Introduction

Positional vertigo is one of the most frequent Reasons for consultation in patients presenting with dizziness and balance disorders. Benign paroxysmal positional vertigo (BPPV) is the most common cause of vertigo worldwide, with a lifetime prevalence of 2.4%, a 1-year prevalence of 1.6%, and a 1-year incidence of 0.6% [1,2]. Although BPPV is generally considered a benign peripheral vestibular disorder, several central nervous system pathologies may present with positional symptoms that closely mimic BPPV, creating important diagnostic challenges for clinicians [3].

The pathophysiology of BPPV is commonly explained by the displacement of otoconia into one of the semicircular canals [4]. These particles, which are heavier than the surrounding endolymph, move according to head position relative to gravity and generate abnormal stimulation of the cupula, resulting in vertigo and positional nystagmus [4]. This mechanism accounts for the characteristic latency, limited duration, fatigability, and direction-changing properties of BPPV nystagmus, as well as the effectiveness of repositioning maneuvers [4].

To standardize diagnosis, the Bárány Society established diagnostic criteria for BPPV based on recurrent episodes of positional vertigo or dizziness triggered by changes in head position and associated with characteristic positional nystagmus [5]. The diagnosis primarily relies on bedside positional testing, including the Dix–Hallpike maneuver for the posterior and anterior semicircular canals and the supine roll test for the horizontal canal [6]. In patients presenting with a typical clinical picture, neither brain nor inner ear imaging is routinely required [7].

However, not all positional vertigo is peripheral in origin. Certain central lesions involving the cerebellum, brainstem, or vestibulocerebellar pathways may produce positional vertigo and nystagmus that resemble BPPV. In these situations, clinicians should carefully search for clinical features inconsistent with a typical peripheral disorder, including persistent positional nystagmus, downbeat or direction-changing nystagmus, fixation-resistant nystagmus, associated oculomotor abnormalities, cerebellar signs, prolonged symptoms, or failure to respond to appropriate repositioning maneuvers [8,9].

Central positional nystagmus (CPN) is defined as nystagmus triggered by a change in head position relative to gravity and resulting from pathology affecting central vestibular pathways [10]. CPN may be paroxysmal or persistent and has been associated with a wide range of disorders, including ischemic stroke, posterior fossa tumors, demyelinating disease, cerebellar degeneration, autoimmune and paraneoplastic syndromes, as well as vestibular migraine [8,[11], [12], [13]]. Because its clinical presentation may overlap with that of BPPV, CPN is frequently considered a diagnosis of exclusion in patients presenting with positional vertigo [14].

When a central origin is suspected, neuroimaging becomes essential. Importantly, routine head computed tomography and standard screening magnetic resonance imaging (MRI) protocols may be insufficient to evaluate the posterior fossa and can fail to detect small ischemic lesions, subtle cerebellar abnormalities, or neurovascular conflicts. An appropriate imaging protocol should include diffusion-weighted imaging (DWI) with apparent diffusion coefficient (ADC) mapping to identify acute infarcts, particularly within the brainstem and cerebellum. High-resolution thin-slice posterior fossa sequences, including 3-dimensional T2-weighted acquisitions and heavily T2-weighted sequences such as constructive interference in steady state (CISS) or fast imaging employing steady-state acquisition (FIESTA), are recommended for detailed assessment of the brainstem, dorsal cerebellar vermis, floor of the fourth ventricle, cerebellopontine angle, and cranial nerves [8,15]. These dedicated sequences substantially improve the detection of small tumors, neurovascular compression syndromes, demyelinating lesions, and other subtle central causes of positional vertigo.

Recent recommendations from the French Society of Ear, Nose, and Throat (ENT) Medicine emphasize that patients presenting with atypical positional nystagmus should be carefully evaluated before undergoing repositioning maneuvers [16]. In particular, horizontal ageotropic positional nystagmus and predominantly downbeat positional nystagmus should raise suspicion for a possible central neurological disorder [16]. In such cases, further neurological assessment and dedicated MRI evaluation are strongly recommended.

Although central positional vertigo is well recognized in the literature, it remains frequently misdiagnosed as BPPV in routine clinical practice because of overlapping positional symptoms. Delayed recognition may result in inappropriate repositioning maneuvers, missed neurological diagnoses, and delays in appropriate treatment. The present case series illustrates a spectrum of central disorders initially suspected to be BPPV and highlights the clinical red flags and imaging findings that should prompt further neurological investigation. Particular emphasis is placed on the role of dedicated posterior fossa MRI protocols in establishing the correct diagnosis and avoiding diagnostic pitfalls.

## Case series

We present 6 clinical cases of patients who consulted vestibular physiotherapists at the request of their general practitioner or ENT specialist for positional vertigo initially diagnosed as BPPV. Inclusion criteria for this case series were: (1) referral for presumed BPPV by a general practitioner or ENT specialist; (2) presence of positional vertigo as the main presenting symptom; (3) identification of atypical clinical findings during vestibular assessment suggesting a possible central origin; and (4) availability of neuroimaging that established or supported the final diagnosis. Patients with typical BPPV successfully treated by repositioning maneuvers and without atypical neurological or oculomotor signs were not included.

The clinical workflow followed a stepwise approach. All patients underwent a detailed history, positional testing (Dix–Hallpike and/or supine roll test), and a comprehensive vestibular and neurological examination including ocular motor assessment. When findings were inconsistent with typical BPPV, such as persistent positional nystagmus, downbeat nystagmus, gaze-evoked nystagmus, skew deviation, abnormal smooth pursuit, cerebellar signs, refractory symptoms, or atypical symptom chronology, patients were referred for specialist neurological or otoneurological evaluation. Brain MRI was subsequently performed to investigate potential central causes of positional vertigo and to guide further management.

The presentation of these cases allows us to review the differential diagnoses of BPPV and the identification of red flags. Particular attention is given to the contribution of radiological imaging in establishing the final diagnosis and in differentiating peripheral vestibular disorders from central nervous system pathologies. We systematically describe the clinical history of each of these patients, their clinical examination, and the origin of their positional vertigo. For each case, we also detail the imaging workup performed, including brain MRI and other relevant radiological examinations, as well as the specific imaging findings that led to the diagnosis. MRI protocols and sequences used to evaluate the posterior fossa and brainstem are highlighted when relevant. All patients gave their consent for the dissemination of their clinical history and signed the informed consent form.

### Case 1

**Fig. 1:**
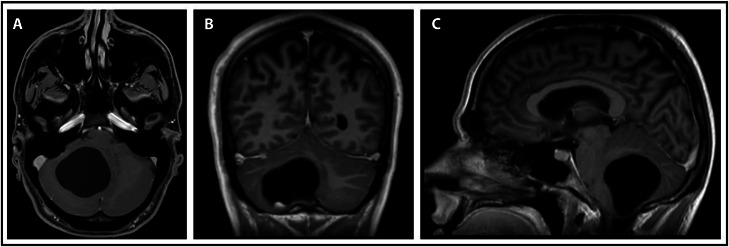
Contrast-enhanced T1-weighted brain MRI showing a predominantly cystic right cerebellar infratentorial mass consistent with a hemangioblastoma. (A) Axial image demonstrates a large cystic lesion centered on the right cerebellar hemisphere, exerting mass effect on the cerebellum and fourth ventricle. (B) Coronal image shows an enhancing mural nodule along the inferior aspect of the cystic lesion, a hallmark imaging feature of hemangioblastoma. (C) Sagittal image illustrates the craniocaudal extent of the lesion within the posterior fossa without evidence of secondary obstructive hydrocephalus.

A 32-year-old French male patient was referred by his general practitioner for BPPV. The patient complained of positional vertigo, accompanied by nausea without vomiting, tinnitus, or headaches. The clinical examination revealed a persistant, downbeat and rightward oblique nystagmus, on the bilateral Dix-Hallpike maneuvers. The nystagmus was not inhibited by visual fixation. The Romberg test and cerebellar tests were normal, as was the Head Impulse Test (HIT). However, the cover test revealed a vertical misalignment and the patient exhibited gaze-evoked nystagmus. Given these findings, a rapid ENT consultation was requested. In the meantime, the patient’s condition worsened with the onset of headaches and cerebellar signs. Brain MRI was performed using a dedicated posterior fossa protocol including axial and sagittal T1-weighted and T2-weighted sequences, FLAIR, DWI with apparent diffusion coefficient (ADC) mapping, susceptibility-weighted imaging, and post-gadolinium contrast-enhanced T1-weighted sequences. Imaging demonstrated a well-defined cystic lesion with an enhancing mural nodule in the right cerebellar hemisphere, associated with mild mass effect on adjacent cerebellar structures. No hydrocephalus was identified on MRI (Fig. 1). These radiological features were highly suggestive of cerebellar hemangioblastoma and allowed prompt neurosurgical referral. The patient underwent surgery within the month for tumor resection, which led to a complete resolution of his symptoms post-surgery.

Hemangioblastomas are benign, highly vascularized tumors primarily located in the central nervous system [17]. They are benign tumors originating from blood vessels, making up 0.5% of all intracranial tumors and 7%-12% of posterior fossa tumors in adults [18,19]. Although benign, these lesions can lead to serious complications and potentially be life-threatening especially in case of intratumoral bleeding. Symptoms primarily arise due to the mass effect of the primary tumor, as well as the development of cysts around the tumor and peritumoral edema, which affects approximately 70%-75% of symptomatic cerebellar tumors [20,21]. Studies on cerebellar hemangioblastomas [20,22] have shown that the most commonly reported symptoms were gait ataxia (12%-64%), dysmetria (29%-64%), headaches (12%-75%), and nausea/vomiting (8%-34%).

On MRI, cerebellar hemangioblastomas typically appear as cystic lesions with an enhancing mural nodule and prominent vascularity. Contrast-enhanced MRI is considered the imaging modality of choice for diagnosis and preoperative planning, as it accurately delineates tumor boundaries, vascular supply, and associated cystic components. In cases of unexplained positional vertigo with central ocular motor signs, early MRI of the posterior fossa is essential to exclude such space-occupying lesions and guide appropriate management [23].

### Case 2

An 18-year-old French male patient was referred by his general practitioner for BPPV. For several months, he had been experiencing positional vertigo occurring in the morning upon waking when he sat up in bed, as well as in the morning while in certain positions related to his work as a heating technician. These vertigo episodes, lasting from several minutes to several hours, were fluctuating, with periods of remission lasting several days followed by recurrences. The patient reported vertical eye misalignment since childhood and had previously suffered from migraines. He also mentioned fluctuating headaches over the past few weeks, which he had difficulty characterizing. During the clinical examination, while the patient was experiencing vertigo, a very subtle spontaneous downbeat nystagmus was observed, persistent during all positional tests (supine roll test and Dix-Hallpike). The rest of the clinical examination was normal, and no signs of BPPV were found. Brain MRI was performed and particular attention was paid to the cerebellum, brainstem, and cerebellopontine angle regions. No structural lesions, demyelinating plaques, ischemic changes, or posterior fossa masses were identified. The patient's complaints matched the criteria for vestibular migraine as defined by the Barany Society, particularly because his symptoms could not be explained by another vestibular disorder, and he is known to have a history of migraines.

The absence of abnormalities on MRI was essential to exclude structural central causes of positional vertigo, thereby supporting the diagnosis of vestibular migraine, which remains primarily a clinical diagnosis after exclusion of radiologically detectable pathologies.

Vestibular migraine would affect between 1% and 2.7% of the general population [24,25]. Diagnostic criteria have been established for vestibular migraine [26]. They include at least 5 episodes with vestibular symptoms of moderate or severe intensity, lasting from 5 minutes to 72 hours. Patients must have a current or past history of migraine with or without aura, in accordance with the International Classification of Headache Disorders (ICHD-3). Additionally, at least 50% of vestibular episodes must be accompanied by 1 or more migraine features, such as headache with at least 2 of the following criteria: unilateral location, pulsating quality, moderate or severe intensity, aggravation by routine physical activity, photophobia and phonophobia, or visual aura. Finally, these symptoms should not be better explained by another vestibular or ICHD diagnosis. It is also noted that approximately 10% of patients with vestibular migraine may report positional vertigo [26]. To differentiate vestibular migraine positional vertigo from BPPV, it is important to consider, in addition to the clinical context, the nystagmus. In vestibular migraine, the positional nystagmus is generally persistent, with a speed typically low to moderate, rarely exceeding 30°/s [27]. In contrast, in BPPV, the nystagmus is much faster, with speeds reaching up to 275°/s [27]. Symptomatic episodes are often shorter in vestibular migraine, lasting from a few minutes to a few days [26], in contrast to those of BPPV, which can last several weeks.

### Case 3

A 67-year-old French female patient was referred by her ENT specialist for BPPV. She had experienced fluctuating positional vertigo over time, with more than 10 episodes occurring over the past 30 years, each lasting a few weeks. During each episode, she underwent vestibular rehabilitation, including Semont and Epley maneuvers, but these interventions had shown little effect. According to the patient, the vertigo would spontaneously resolve over time. During the most recent episode, 6 maneuvers were performed by a vestibular physiotherapist and 1 by her ENT specialist, with no significant improvement. The patient was then referred to another vestibular physiotherapist. Initial brain MRI had been interpreted as normal.

On clinical examination, the presentation and symptoms were consistent with BPPV: positional rotational vertigo during the left Dix-Hallpike, associated with a left torsional nystagmus in the same direction as left posterior semicircular canal BPPV (a left torsional nystagmus with a up-beating component), inhibited by visual fixation. However, the nystagmus had an unusual duration and did not reverse when returning to the seated position. The patient was highly symptomatic and exhibited a significant vasovagal reaction.

**Fig. 2:**
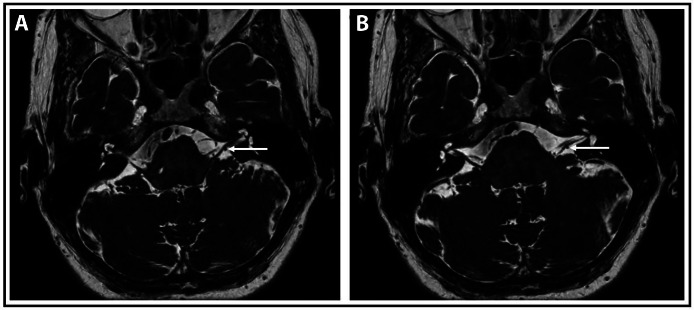
High-resolution MRI demonstrating neurovascular compression of the left vestibular nerve. (A and B) Heavily T2-weighted CISS/FIESTA images show close contact between the anterior inferior cerebellar artery (AICA) and the vestibulocochlear nerve within the cerebellopontine angle cistern (arrows). These high-resolution sequences provide excellent contrast between cerebrospinal fluid, cranial nerves, and vascular structures, facilitating identification of neurovascular conflict consistent with vestibular paroxysmia.

A second targeted radiological review using high-resolution MRI sequences of the cerebellopontine angle was performed. Heavily T2-weighted 3-dimensional sequences such as constructive interference in steady state (CISS/FIESTA) and T2-weighted imaging with fine slices enabled visualization of the neurovascular conflict between the anterior inferior cerebellar artery and the left vestibular nerve within the cerebellopontine angle (Fig. 2). These sequences are considered essential for detecting vascular compression of cranial nerves and are more sensitive than conventional MRI protocols.

The identification of this neurovascular compression on dedicated MRI sequences allowed a revision of the initial diagnosis and supported the diagnosis of vestibular paroxysmia, explaining the patient’s recurrent positional vertigo refractory to repositioning maneuvers.

The type of neurovascular compression syndrome, also called vestibular paroxysmia, is considered a rare disease, affecting fewer than 1 in 2000 people, even though its prevalence remains unknown [28]. A study conducted in a tertiary care center among more than 45,234 patients suffering from vertigo and dizziness estimated the relative frequency of vestibular paroxysmia to be around 4% [29]. It is believed that the brief episodes of vertigo are triggered by ephaptic discharges, that is, abnormal paroxysmal transmissions between neighboring, partially demyelinated axons [28]. The neurovascular conflict would occur in this area, at the level of the central myelin (oligodendroglia), near the “transition zone” [30]. Diagnostic criteria have been established to better define this condition, including at least 10 spontaneous episodes of vertigo, whether spinning or not, lasting less than 1 minute, with a stereotypical phenomenology specific to the patient, a response to treatment with carbamazepine/oxcarbazepine, and no other diagnosis explaining the symptoms [28]. Several decades ago, microvascular decompression surgeries of the eighth nerve were attempted in patients with an association between this anatomical feature and symptoms of positional vertigo resistant to conventional medication treatments and habituation therapy [31]. In almost all of these patients (8 out of 9), symptoms improved after the surgery [31].

MRI of the cerebellopontine angle with high-resolution 3-dimensional T2-weighted sequences is considered the imaging modality of choice for detecting vestibulocochlear nerve vascular compression. Radiological identification of neurovascular conflict is crucial in patients with atypical or treatment-resistant positional vertigo, as it guides medical therapy and may support consideration of microvascular decompression in selected cases [32].

### Case 4

A 59-year-old French male patient had been complaining for the past 3 years, of persistently occuring episodes of positional rotational vertigo when tilting his head forward or backward. These episodes lasted only 2-3 seconds and could be accompanied by a sensation of drunkenness afterwards during head movements. These symptoms were also present when lying on his back, which led him to sleep with his head elevated. On clinical examination, the Romberg, the Fukuda, and the finger-to-nose test were normal, and ocular motility was normal. However, in the dark, a horizontal left-beating nystagmus (grade I follows Alexander’s law) was observed, which increased after the head-shaking test. During the right Dix-Hallpike test, a violent right torsional, up-beating nystagmus appeared, which then became horizontal and poorly exhaustible. Similarly, during the left Dix-Hallpike test, a geotropic nystagmus was observed, which was poorly exhaustible and barely felt by the patient. Additionally, a downbeat nystagmus appeared occasionally without any specific movement. The hypothesis was left lateral canal BPPV and possibly right posterior canal BPPV, but with numerous atypical features (chronology, no inversion upon returning to the seated position, persistent nature of the symptoms).

A week later, a videonystagmography was performed, revealing hypometric eye movements when looking upward exclusively, with an inability to maintain this position and the presence of an upbeat nystagmus. Significant impairment of vertical smooth pursuit was noted (very saccadic), and optokinetic nystagmus was absent. Furthermore, no spontaneous nystagmus was observed in light or darkness, except for a slight upper-left oblique nystagmus unaffected by fixation. The rest of the examination was normal. These tests ruled out the hypothesis of lateral canal BPPV and suggested a central pathology.

**Fig. 3:**
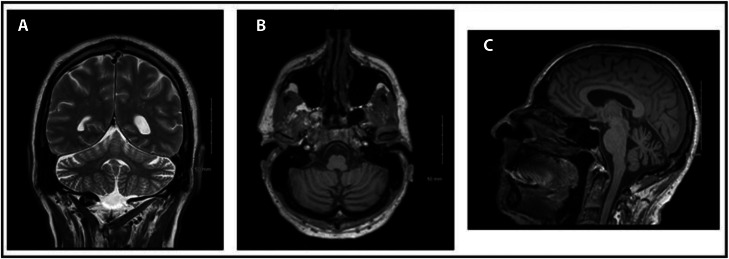
Brain MRI demonstrating cerebellar atrophy. (A) Coronal T2-weighted image showing diffuse cerebellar volume loss with widening of cerebellar folia and fissures. (B) Axial T2-weighted image confirming bilateral cerebellar hemispheric atrophy without focal signal abnormality. (C) Sagittal T1-weighted image demonstrating vermian involvement and enlargement of surrounding cerebrospinal fluid spaces. The absence of a focal lesion favors a degenerative cerebellar process rather than a neoplastic or vascular etiology.

Brain MRI demonstrated diffuse cerebellar atrophy involving the cerebellar vermis and hemispheres. Imaging demonstrated diffuse cerebellar volume loss, predominantly involving the cerebellar vermis and hemispheres, with enlargement of cerebellar folia and widening of the cerebellar fissures (Fig. 3). No focal mass lesion or acute ischemic changes were identified. These radiological findings were consistent with cerebellar atrophy and supported the hypothesis of a chronic degenerative or structural central vestibular disorder.

Three years later, the clinical picture had evolved, showing ocular pursuit dysmetria, dysarthria, and dysmetria predominantly in the left upper limb. Additionally, the patient was unable to walk a narrow line, the initial vertigo was still present, and the patient reported lower limb hypertonia without spasticity. Deep tendon reflexes were normal, and there were no sensory disorders or pyramidal rigidity.

Cerebellar atrophy can be caused by several factors, including genetic diseases [33], trauma [34], infections, or neurodegenerative diseases such as multiple sclerosis [35] or Parkinson's disease [36]. The symptoms of this disease, like its causes, are quite broad and are directly related to the diseases or pathology that triggered it. The most common symptoms of cerebellar atrophy include gait ataxia, limb ataxia, cerebellar dysarthria, and oculomotor signs such as ocular dysmetria, saccadic pursuit, sustained gaze-evoked nystagmus, and positional downbeat nystagmus [37].

MRI is the reference imaging modality for assessing cerebellar atrophy, allowing accurate visualization of cerebellar volume loss and exclusion of other structural posterior fossa abnormalities. In patients presenting with atypical positional vertigo and central oculomotor signs, early MRI evaluation is crucial to identify degenerative cerebellar disorders and guide neurological follow-up and management [38].

### Case 5

**Fig. 4:**
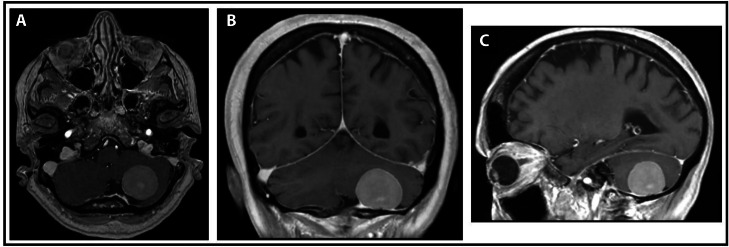
Contrast-enhanced T1-weighted brain MRI images in the axial (A), coronal (B), and sagittal (C) planes, respectively. These images show a well-defined, enhancing left cerebellar extra-axial mass consistent with a meningioma, associated with mild mass effect on the vermis while the fourth ventricle remains patent.

An 85-year-old French female patient was referred by her general practitioner for BPPV, knowing that the patient had been diagnosed with a cerebellar meningioma (left extra-axial cerebellar mass) 4 years earlier (Fig. 4). The patient had positional rotational vertigo lasting 15 seconds during the left Dix-Hallpike maneuver with a typical upbeat, left torsional nystagmus, suggestion BPPV. This nystagmus had a 2-second latency before appearing on the left Dix-Hallpike and reversed upon returning to the seated position. Once it stopped, it was replaced by an asymptomatic ageotropique horizontal nystagmus on both sides of the Dix-Hallpike and head-roll test. Given the left posterior semicircular canal BPPV, an Epley maneuver was performed, and a week later, the positional vertigo had disappeared, as well as the nystagmus. The physiotherapist did not treat the ageotropic nystagmus as a cupulolithiasis of a lateral semicircular canal because it was atypical and, in his opinion, related to the cerebellar tumor mass. The patient had a significantly widened base of support, normal cerebellar tests, a negative cover test, regular headaches, urinary incontinence, and an abnormal gait. The patient was referred to a neurosurgeon to reassess the meningioma, which was exerting a slight mass effect on the vermis structures.

Follow-up brain MRI was performed to reassess the known lesion and evaluate potential progression or mass effect. MRI demonstrated a well-circumscribed extra-axial enhancing lesion in the left posterior fossa consistent with a cerebellar meningioma, associated with mild compression of adjacent cerebellar parenchyma and vermian structures without significant perilesional edema or hydrocephalus.

Although meningiomas are typically benign and encapsulated tumors with few genetic aberrations, their intracranial location can often lead to serious and potentially fatal consequences [39]. Meningiomas represent nearly one-third of all central nervous system tumors, with an incidence of approximately 7.10 per 100,000 people [40]. Among older adolescents and young adults (15-39 years) and individuals aged 40 years or older, the age-adjusted incidence of cerebellar tumors is 10.71 and 40.10 per 100,000 people [41]. Meningiomas located in the posterior cranial fossa make up about 9% of all intracranial meningiomas [42]. Motor impairments resulting from an affected cerebellum include ataxia and dysmetria, with ataxia generally associated with walking difficulties, and dysmetria related to the loss of coordinated limb movements [43].

Contrast-enhanced MRI is the imaging modality of choice for the evaluation and follow-up of posterior fossa meningiomas, allowing precise assessment of tumor size, extra-axial location, dural attachment, and mass effect on adjacent cerebellar and brainstem structures. In patients presenting with vertigo and known posterior fossa tumors, MRI plays a crucial role in distinguishing coincidental peripheral vestibular disorders from symptoms related to tumor progression or cerebellar compression [44].

### Case 6

**Fig. 5:**
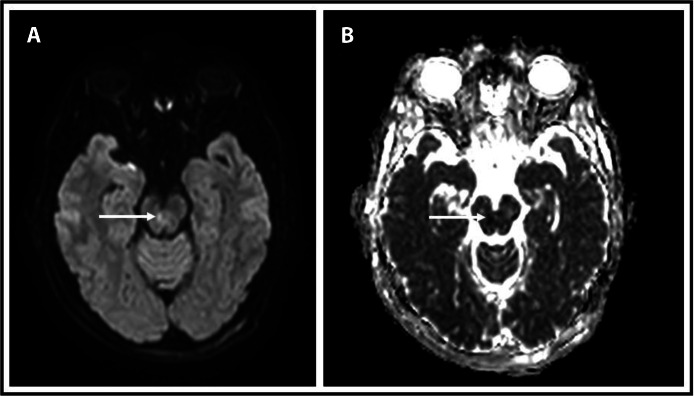
Brain MRI demonstrating an acute right midbrain ischemic infarction. (A) Diffusion-weighted imaging (DWI) demonstrates focal hyperintensity within the right midbrain (arrow), indicating restricted diffusion. (B) Corresponding ADC map shows signal hypointensity at the same location (arrow), confirming true diffusion restriction and supporting the diagnosis of acute ischemic stroke. The DWI–ADC mismatch is characteristic of recent infarction and allows early detection of posterior circulation ischemic lesions that may be occult on conventional imaging.

A 54-year-old French female patient was referred to a physiotherapist by her general practitioner for a specialized assessment due to positional vertigo that appeared within a month following her right midbrain stroke. Initially, the patient went to the emergency department after experiencing a loss of consciousness for several minutes, accompanied by persistent rotary vertigo. Brain MRI was performed using an acute stroke protocol. An hyperintense lesion was demonstrated on DWI with corresponding low ADC values and absence of FLAIR signal abnormality, consistent with a hyperacute ischemic infarct of the right midbrain (Fig. 5). In the days and weeks that followed, the continuous rotary vertigo gradually subsided, leaving only positional vertigo.

In this context, the general practitioner referred the patient to a physiotherapist specialized in vestibular rehabilitation for an assessment and, if necessary, a liberatory maneuver for BPPV. During the clinical examination, the patient exhibited saccadic smooth pursuit and a spontaneous right horizonto-rotatory nystagmus of grade III follows Alexander’s law, continuous in the right eye but fluctuating in the left eye, likely linked to a lesion of the medial longitudinal fasciculus. The rest of the clinical examination was normal, including the Dix-Hallpike test and the head supine roll test on both sides, which only revealed spontaneous nystagmus without any signs suggestive of BPPV. Consequently, no liberatory maneuver, such as Epley or Baloh-Lempert, was performed.

Follow-up MRI is recommended in such cases to monitor lesion evolution and to detect possible secondary changes within the brainstem or cerebellum that may contribute to persistent vestibular symptoms. MRI remains the reference imaging modality for evaluating posterior circulation strokes and their sequelae.

Almost one-third of ischemic strokes affect the vertebrobasilar system [45]. First ischemic vertebrobasilar strokes affect the pons (27%) more commonly than the medulla (14%) or midbrain (7%) [46]. It is important to keep in mind that it is extremely rare to only observe positional vertigo in the case of a midbrain stroke, due to the high density of anatomical structures in this region [47]. Moreover, significant clinical variability has been observed in acute posterior circulation strokes affecting the midbrain, including hemiplegia, ophthalmoplegia, consciousness disturbances, and gait ataxia [48]. It seems that midbrain strokes with transient rotational vertigo are primarily manifested by lesions in the caudal midbrain tegmentum [47]. Indeed, the MRIs reported by Dieterich and his collaborators reveal lesions that are quite similar to those presented by our patient [47].

Diffusion-weighted MRI is considered the most sensitive imaging technique for detecting acute ischemic lesions of the posterior circulation, including small brainstem infarcts that may not be visible on conventional CT imaging. In patients presenting with acute or subacute vertigo and central oculomotor signs, early MRI with DWI is essential for accurate diagnosis and timely management of ischemic stroke [49].

## Discussion

**Fig. 6:**
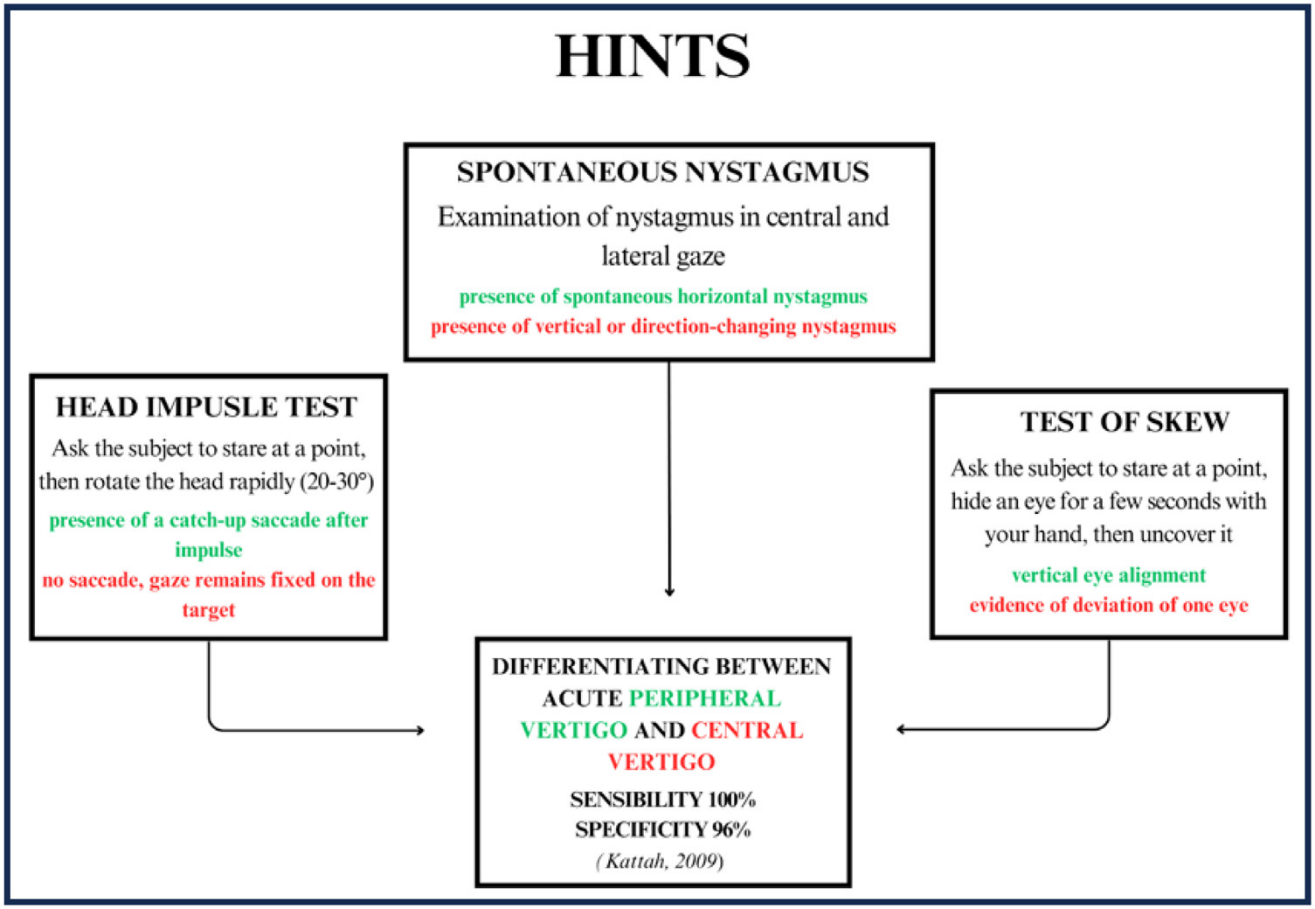
Diagnostic algorithm for the HINTS examination in the evaluation of acute vestibular syndrome in the emergency department.

When receiving a patient in the emergency department for a recent acute vertigo episode (whether positional or not), it is necessary to perform HINTS (Head Impulse test, observation of Nystagmus, and Test of Skew). The HINTS exam is utilized to detect central lesions in acute vestibular syndrome and differentiate them from peripheral diagnostic alternatives, which are more frequent and generally benign [50]. This 3-step bedside test was quickly adopted for its high sensitivity (100%) and specificity (96%), outperforming early diffusion-weighted MRI in stroke diagnosis [50,51]. First, the Head Impulse Test (HIT) is performed to check for the presence of a corrective saccade after the head impulse. The absence of saccades suggests a central origin. Next, the presence of spontaneous horizontal nystagmus in lateral gaze (without spontaneous nystagmus in central gaze) or pure vertical nystagmus in frontal gaze also points toward a central cause. Finally, a vertical deviation of 1 eye during the test of skew, known as skew deviation, strongly suggests a central lesion. All these elements are illustrated in Figure 6.

However, although HINTS demonstrates excellent diagnostic performance when performed by trained clinicians, neuroimaging, particularly brain MRI with diffusion-weighted imaging, remains essential to confirm the diagnosis, identify the precise anatomical lesion, and guide management in suspected central vertigo.

First, the HIT is performed to check for the presence of a corrective saccade after the head impulse. The absence of saccades suggests a central origin. Next, the presence of spontaneous horizontal nystagmus in lateral gaze (without spontaneous nystagmus in central gaze) or pure vertical nystagmus in frontal gaze also points toward a central cause. Finally, a vertical deviation of 1 eye during the test of skew, known as skew deviation, strongly suggests a central lesion. When any of these central signs are identified, urgent brain MRI focusing on the posterior fossa and brainstem is recommended to detect ischemic, tumoral, demyelinating, or degenerative lesions [52].

The HINTS can be complemented by a cerebellar test such as the finger-to-nose test, which, when abnormal, is a major risk factor for predicting cerebrovascular events in patients with isolated dizziness, with a notable increase in diagnostic performance [53]. Therefore, we recommend using the finger-to-nose test in the emergency department to assess the need for further diagnostic evaluation [53]. A 2019 study by Oostema even highlighted that paramedics trained in the finger-to-nose test were more likely to identify posterior strokes compared to untrained paramedics [54]. In this diagnostic algorithm, early MRI evaluation is essential when central pathology is suspected, as it allows visualization of posterior circulation strokes, cerebellar degeneration, and posterior fossa tumors that may present with isolated vertigo.

Once central neurological tests have been performed and found to be reassuring, a Dix-Hallpike test can be conducted to determine whether the patient's positional vertigo is due to BPPV, as it appears to be the reference test for diagnosing this condition [55]. This test allows for the identification of BPPV of the posterior semicircular canal if the previously mentioned diagnostic criteria are also present [5]. Regarding the diagnosis of BPPV of the horizontal semicircular canal, the test of choice is the supine roll test [5]. In typical BPPV presentations with classic clinical findings, imaging is generally not required. However, in atypical or refractory cases, MRI is recommended to exclude central causes that may mimic peripheral vestibular disorders [8].

In case of doubt or the presence of a central sign in positional vertigo, it is best to refrain from performing maneuvers on the patient and to seek the opinion of a general practitioner, an ENT specialist, a neurologist, or an emergency physician for a more in-depth evaluation, as was done in each of these clinical cases. Radiological evaluation, particularly brain MRI with dedicated posterior fossa sequences, should be performed promptly when central vertigo is suspected, as it plays a decisive role in establishing the diagnosis and guiding therapeutic management.

An important diagnostic distinction should be made between true central positional vertigo and situations in which a peripheral vestibular disorder coexists with a central nervous system lesion. In true central positional vertigo, positional vertigo and nystagmus arise directly from dysfunction of central vestibular pathways, most commonly involving the cerebellum or brainstem. In contrast, some patients may fulfill the diagnostic criteria for BPPV while also harboring an unrelated central lesion [56].

Most patients in our series belonged to the first category, as their positional symptoms were ultimately explained by central pathologies including cerebellar hemangioblastoma, vestibular migraine, vestibular paroxysmia, cerebellar atrophy, and midbrain ischemic stroke. Conversely, Case 5 illustrates a coexistence mechanism, in which a typical posterior canal BPPV responded to an Epley maneuver despite the presence of a cerebellar meningioma. This case highlights that the identification of a central lesion on neuroimaging should not automatically lead clinicians to attribute all positional symptoms to a central mechanism. Rather, careful analysis of positional nystagmus characteristics, response to repositioning maneuvers, and associated neurological findings remain essential to distinguish true central positional vertigo from concomitant peripheral vestibular disorders.

To date, many patients with neurological disorders are still mistakenly referred to vestibular physiotherapists by general practitioners. On the other hand, since specialization in vestibular rehabilitation is not a regulatory requirement for managing vertigo patients, many non-specialized physiotherapists still perform liberatory maneuvers for BPPV without conducting an appropriate clinical examination. It is essential to conduct a proper clinical examination to accurately assess patients, understand the differential diagnoses of BPPV, and avoid diagnostic errors. In this context, systematic consideration of neuroimaging when atypical signs are present is essential to avoid misdiagnosis and delays in the management of central nervous system pathologies.

We collected, through a questionnaire, the experiences of several French physiotherapists specialized in vestibular rehabilitation and members of the French and Francophone Society of Vestibular Rehabilitation (SFFRV). Many of them have also treated patients referred for BPPV, while the positional vertigo experienced by these patients actually had another origin, such as an Arnold Chiari malformation or a vascular origin in the posterior fossa. Arnold-Chiari malformations are rare abnormalities characterized by downward displacement of the cerebellar tonsils and the brainstem through the foramen magnum [57]. It is important to consider Chiari malformations in the differential diagnosis of recurrent syncope [57]. These positional syncopal episodes are attributed either to compression of the midbrain's ascending reticular system or to vascular compromise, such as vertebrobasilar artery compression or hypotension [58]. A case of Chiari malformation reportedly had its syncopal episodes disappear after surgery for the malformation [58]. Another clinical case of a 51-year-old patient with Arnold-Chiari type I malformation, initially diagnosed as benign paroxysmal positional vertigo of the posterior semicircular canal, has been reported in the literature [59]. It is therefore very important to recognize Arnold-Chiari type I malformation in the differential diagnosis of vertigo cases in adults [59]. High-resolution MRI of the craniovertebral junction is the imaging modality of choice for diagnosing Chiari malformations and assessing associated brainstem or cerebellar compression [60].

It is also important to suspect a vascular origin for an isolated rotational vertigo, as can be the case in a vertebral artery dissection [61]. Magnetic resonance angiography (MRA) or computed tomography angiography (CTA) may be required in such cases to evaluate the vertebrobasilar circulation and detect vascular abnormalities.

When an atypical positional vertigo of BPPV presents, it is crucial not to take the risk of performing a maneuver without ensuring whether its origin is central or peripheral. Indeed, certain central neurological conditions, such as a posterior fossa tumor, can lead to cerebellar tonsillar herniation, potentially resulting in life-threatening complications [62]. A case has been reported in the literature involving a middle-aged patient with depressive disorders, who presented to a psychiatric hospital due to the worsening of her symptoms, including headaches, dizziness, and nausea, over a period of 2 months [63]. The following morning, she was found asleep and was declared dead an hour later. Postmortem tomography and autopsy revealed a large cyst in the right cerebellar hemisphere (a hemangioblastoma, like in our clinical case 1), accompanied by hydrocephalus and transforaminal herniation. The cause of death was attributed to brainstem compression caused by obstructive hydrocephalus secondary to the cystic hemangioblastoma of the cerebellum. These cases remain fortunately rare, but it is important to be aware of them, as it can be easily imagined that an Epley or Semont liberatory maneuver could worsen the process of tonsillar herniation. Early MRI evaluation of posterior fossa symptoms is therefore essential to detect space-occupying lesions and prevent potentially fatal complications.

The present case series illustrates the diversity of imaging patterns encountered in central causes of positional vertigo. As summarized in Table 1, MRI findings varied according to the underlying pathophysiological mechanism, ranging from structural lesions and ischemic infarction to degenerative disorders, neurovascular compression, and conditions with normal structural imaging such as vestibular migraine. This comparative overview highlights the value of tailored neuroimaging protocols in patients presenting with atypical positional vertigo and supports the integration of imaging findings into the diagnostic reasoning process.

**Table 1:** Comparative MRI characteristics of central causes of positional vertigo identified in this case series.

Etiology	MRI findings	Key sequences	Main red flags
Cerebellar hemangioblastoma	Cystic lesion with enhancing mural nodule	T1+C, T2, FLAIR	Downbeat nystagmus, skew deviation
Vestibular migraine	Normal MRI	Standard MRI	Migraine history, persistent low-velocity nystagmus
Vestibular paroxysmia	Neurovascular conflict AICA-CN VIII	CISS/FIESTA	Recurrent brief attacks, treatment resistance
Cerebellar atrophy	Vermian and hemispheric atrophy	T1, T2	Oculomotor abnormalities
Cerebellar meningioma	Enhancing extra-axial mass	T1+C	Cerebellar signs, gait disorder
Midbrain stroke	DWI restriction	DWI/ADC	Acute onset, central ocular signs

ADC, apparent diffusion coefficient; AICA, anterior inferior cerebellar artery; BPPV, benign paroxysmal positional vertigo; C, contrast; CISS, constructive interference in steady state; CN VIII, vestibulocochlear nerve; DWI, diffusion-weighted imaging; FIESTA, fast imaging employing steady-state acquisition; MRI, magnetic resonance imaging.

In conclusion, clinicians evaluating patients with positional vertigo should maintain a high index of suspicion for a central cause when the clinical presentation is inconsistent with typical BPPV. Features warranting further investigation include unusual positional nystagmus patterns (particularly persistent downbeat, direction-changing, or fixation-resistant nystagmus), associated oculomotor abnormalities, cerebellar signs, prolonged or recurrent symptoms, and poor or absent response to appropriate repositioning maneuvers. In such situations, patients should be referred for specialist assessment and neuroimaging rather than undergoing repeated therapeutic maneuvers.

This case series also highlights that the diagnostic yield of imaging depends on the quality of the imaging protocol. Routine head CT and standard screening brain MRI examinations may fail to identify small posterior fossa lesions, brainstem infarcts, subtle cerebellar abnormalities, or neurovascular conflicts. Dedicated posterior fossa MRI protocols, including DWI with ADC mapping and high-resolution thin-slice 3-dimensional T2-weighted or CISS/FIESTA sequences, are often required to adequately evaluate central positional vertigo mimics.

This case series emphasizes the importance of recognizing clinical red flags suggestive of central positional vertigo. When atypical features are present, neuroimaging can facilitate timely diagnosis, guide management, and prevent potentially serious diagnostic errors.

## Author contributions

Quentin Legois: Conceptualization, Analysis, Writing – original draft. Paul Fargeas: Conceptualization, Analysis, Writing – original draft. Alexandra Weckel: Analysis, Writing – review & editing. Salim Ismail: Analysis, Writing – review & editing. Mikhael Makhoul: Analysis, Writing – review & editing. Pauline Nieto: Writing – review & editing. Charles Edouard Molinier: Writing – review & editing. Olivier Deguine: Writing – review & editing.

## Data availability statement

The data that support the findings of this study are available on request from the corresponding author.

## Patient consent

The authors certify on their honor that written informed consent for the publication of these cases was obtained from the patients.
